# Mechanical Method for Rapid Determination of Step Count Sensor Settings

**DOI:** 10.3390/bioengineering11060547

**Published:** 2024-05-27

**Authors:** Sydney Lundell, Kenton R. Kaufman

**Affiliations:** Mayo Clinic Motion Analysis Laboratory, Rochester, MN 55905, USA; lundell.sydney@mayo.edu

**Keywords:** wearable sensors, remote monitoring, step count, optimization, signal processing

## Abstract

With the increased push for personalized medicine, researchers and clinicians have begun exploring the use of wearable sensors to track patient activity. These sensors typically prioritize device life over robust onboard analysis, which results in lower accuracies in step count, particularly at lower cadences. To optimize the accuracy of activity-monitoring devices, particularly at slower walking speeds, proven methods must be established to identify suitable settings in a controlled and repeatable manner prior to human validation trials. Currently, there are no methods for optimizing these low-power wearable sensor settings prior to human validation, which requires manual counting for in-laboratory participants and is limited by time and the cadences that can be tested. This article proposes a novel method for determining sensor step counting accuracy prior to human validation trials by using a mechanical camshaft actuator that produces continuous steps. Sensor error was identified across a representative subspace of possible sensor setting combinations at cadences ranging from 30 steps/min to 110 steps/min. These true errors were then used to train a multivariate polynomial regression to model errors across all possible setting combinations and cadences. The resulting model predicted errors with an R^2^ of 0.8 and root-mean-square error (RMSE) of 0.044 across all setting combinations. An optimization algorithm was then used to determine the combinations of settings that produced the lowest RMSE and median error for three ranges of cadence that represent disabled low-mobility ambulators, disabled high-mobility ambulators, and healthy ambulators (30–60, 20–90, and 30–110 steps/min, respectively). The model identified six setting combinations for each range of interest that achieved a ±10% error in cadence prior to human validation. The anticipated range of errors from the optimized settings at lower walking speeds are lower than the reported errors of wearable sensors (±30%), suggesting that pre-human-validation optimization of sensors may decrease errors at lower cadences. This method provides a novel and efficient approach to optimizing the accuracy of wearable activity monitors prior to human validation trials.

## 1. Introduction

Physical activity sensors have grown in popularity over the last decade as the cost of the technology has decreased, with one in four Americans using a smartwatch to track their physical activity [1,2]. It is widely understood that physical activity is a direct indicator of systemic health in adults. Physical activity prevents diseases caused by sedentary behavior and has an impact on the social and psychological wellbeing of the individual. With the increased emphasis on personalized medicine, researchers and clinicians have begun exploring the use of wearable sensors to track patient activity [3,4,5]. Commercially available activity sensors, however, fail to meet the requirements for accurate and repeatable step counts, particularly at lower cadences [2,6]. This inaccuracy at lower walking speeds is believed to be due to increased variability in stride time and lower impact accelerations [7,8]. The average age of the population is increasing, and with it the average walking speed is decreasing, highlighting the need for accuracy in activity sensors, particularly at lower walking speeds. Currently, there is no accepted method for identifying a range of appropriate settings in activity monitors prior to human validation trials [9]. Notably, there are no reports of a methodology for pre-validation of sensor settings prior to human validation experiments by the research groups who develop wearable sensors. This gap is addressed in this manuscript, with the goal of accelerating the selection of sensor settings to achieve high research accuracy.

The most common method for validating step counts involves video recording participants as they walk in a laboratory environment, manually counting each step, and then comparing the observed count to the sensor count [10]. Multiple observers may be used, to account for errors in human observation [11,12]. While accurate, these human observations are limited by the range of walking speeds that participants can consistently emulate. Other factors, such as fatigue in the observers and participants, may decrease the accuracy of the baseline step count and walking speed, respectively [13]. To ensure the accuracy of activity-monitoring devices, particularly at slower walking speeds, proven methods must be established to identify suitable settings in a controlled and repeatable manner prior to human validation trials.

The goal of this paper was to develop a novel and reproducible mechanical method for quantifying the error over a range of cadences during pre-validation to identify the sensor setting(s) that may produce the least error. To accomplish this goal, three aims were undertaken: Aim 1: Design and build a mechanical device that can emulate foot contact, and count these artificial steps. Aim 2: Quantify the error over a range of sensor settings. Aim 3: Build a model to identify the sensor settings with the least error over a range of cadences. In this article, we developed a mechanical step-emulating device, which was used to test sensor accuracy at various speeds across a range of unique setting combinations. The resulting errors were used to train a multivariate polynomial regression model. This model was then utilized to determine which sensor settings would be most appropriate for specific walking speed ranges.

## 2. Materials and Methods

### 2.1. Continuous Stepper

**Figure 1:**
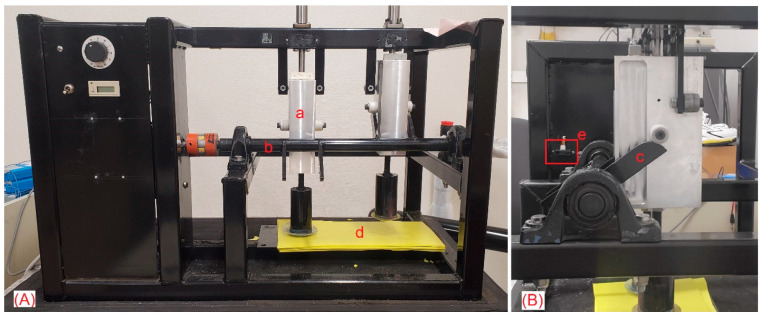
A front view of the continuous impactor is shown in (**A**). (**B**) is a 90-degree rotation looking down the cam (b) toward the distance sensor (e). (a) is one of the two 2.5 kg pistons. Each piston impacts 1 cm of poron foam (d) after being lifted a height of 8 cm by a pair of fins (c).

A mechanical device (Figure 1) was used to lift pistons (1a) 5 cm above a padded platform and drop them at a pre-defined frequency. The rotating cam (1b) had fins (1c) that were 8 cm in length from the center shaft and were situated 180° apart from each other. Every rotation of the shaft resulted in two steps being taken, one from each piston. The pistons (1a) weighed 2.5 kg and were dropped onto a 1 cm thick poron foam (1d) with an impact force of 15 N, or 1.1 g. This impact is consistent with foot impact while walking [8]. A 1/8 hp DC motor was used to drive the cam shaft at speeds ranging from 30 steps/min to 110 steps/min. During data collection, the continuous impactor was encased in a sound-dampening case to minimize noise and protect individuals from the moving parts.

An optical distance sensor (GP2Y0A21YK0F GP2Y0A21, Sharp, Hsin-Chu City, Taiwan) (1e) with a 10–80 cm range was placed proximal to the shafts. Steps were counted according to the change in signal amplitude corresponding to the opposite fin (i.e., when the right piston is lifted, the left fin detects a signal peak near 0.7 V, and a step is counted for the right piston). Total steps were computed from the raw distance sensor data collected at 100 Hz using a National Instruments DAQ USB X Series (National Instruments, Austin, TX, USA). 

### 2.2. Activity Sensor

**Figure 2:**
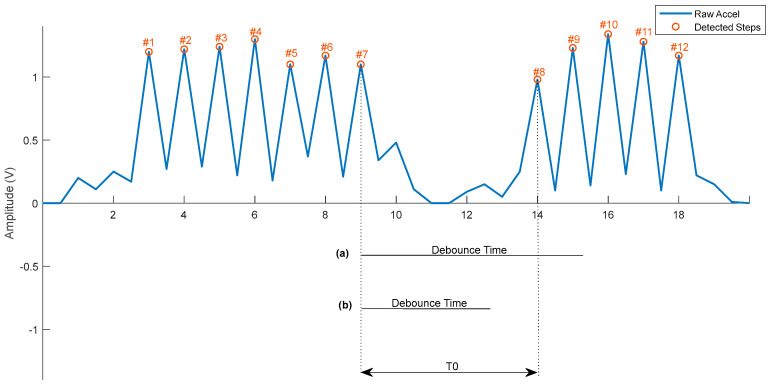
(**a**) denotes an example where 12 steps would be collected for a period of activity. (**b**) depicts the same bout of activity, where only 7 steps have been collected due to a shorter debounce time.

For this study, a commercially available sensor utilizing the LSM6DSL IMU was used [14]. This sensor utilizes a built-in algorithm to determine step count in 10-min increments. The LSM6DSL includes three settings. The “CONFIGU_PEDO_THS_MIN” (threshold) setting determined the minimum detected g’s necessary before steps were counted, with settings ranging from 32 mgs to 996 mgs, totaling 35 options. The “PEDO_DEB_REG” setting contains two settings within the binary registers. The first setting, “Debounce Step”, is the number of steps that need to be detected before the sensor will consider the grouping of impulses to be continuous steps, which ranges from 1 to 7 steps, totaling 7 options. The second setting, “Debounce Time”, ranges from 80 ms to 2480 ms in 80 ms increments, totaling 31 options, and is a window of time after an impulse where another impulse must be detected, or either the previous impulses will be discarded or collection will cease (Figure 2). The cartesian product of these three settings results in a total of 7595 possible combinations.

### 2.3. Signal Processing

**Figure 3:**
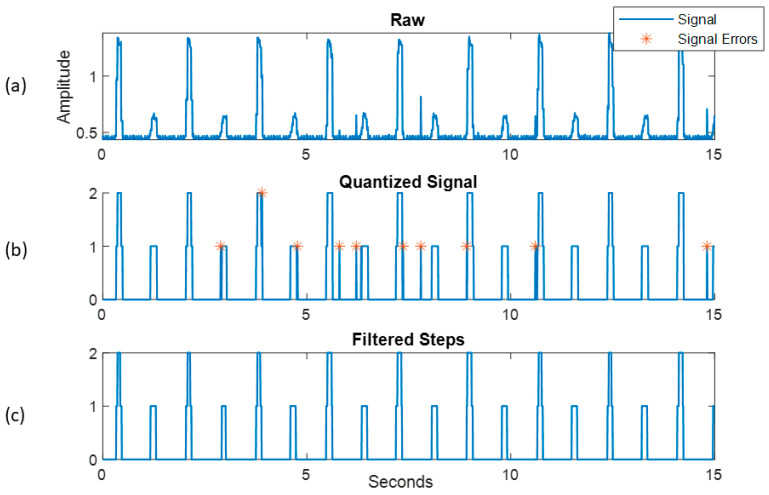
(**a**–**c**) shows the signal progression from a raw signal (**a**) to filtered and quantized (**b**) to finally removing erroneous peaks (**c**) to generate the final step count used and the ground truth for validating the pedometer sensors.

The optical switch located proximal to the cam detected the fins as they rotated, creating a raw signal (Figure 3a). This raw signal was then quantized using a threshold of 1 V, where signals above the threshold were quantized to 1 for a left foot impact and 2 for the right foot impact, with values below 0.5 quantized to 0. Quantizing with only a threshold resulted in erroneous spikes in data that were counted as steps when a peak finding algorithm was applied (Figure 3b). To combat this, the quantized signal was processed using a moving mean filter with a window of three datapoints (0.003 s) to smooth out erroneous spikes in the signal (Figure 3c). Any value not a whole number was removed and replaced with a 0. While this decreased the length of true impacts, it ensured small erroneous spikes caused by sensor noise were not included in the total step count. 

Two sensors were mounted on each piston, for a total of four sensors per trial. Each sensor had a unique orientation (0°, 90°, 180°, 270°) that was randomized for each trial. Sensors were zeroed prior to collection so no erroneous steps were detected during setup. Data were collected in 45 min trials over a range of walking speeds from 30–110 steps/min. A total of 112 data collections, each collecting from four sensors, resulted in a total of 448 setting combinations being tested. This subset of setting combinations was created by sampling the steps, debounce time, and threshold settings every 2, 4, and 5 data points, respectively. The resulting setting combinations were then tested in the continuous stepper at speeds ranging from 30–110 steps/min to determine the sensor error for that combination at a high and low speed.

A final check for erroneous peaks was conducted by looking for an anticipated signal pattern of 1-2-1-2-1-2. If repeated values of 1 or 2 were detected, it suggested an erroneous impact had bypassed the moving mean filter. These erroneous peaks were removed, and the remaining peaks were counted to determine a working total impacts value. To ensure accuracy, the previous filtering method of a moving mean filter was repeated with a window increased by one sample (0.01 s). The resulting impact counts, with repeated 1 s or 2 s removed, were then compared. If the difference was 0, then the resulting total was considered the ground truth for step counts. If the value was not 0, then the process of increasing the window size was repeated until two corrected counts were equal.

Sensor error was then calculated as the delta between the ground truth and sensor steps divided by the total steps from the trial. Error was thusly presented as a percentage from ground truth, with negative error indicating steps being missed and positive error indicating an overcounting of steps.

### 2.4. Regression Model

A total of 448 setting combinations of the possible 7595 were tested, with speeds ranging from 30 steps/min to 110 steps/min. To ensure the magnitude of each setting would not impact the regression outcome, each independent variable (threshold, debounce time, debounce steps, speed) was normalized to a range of 0–1.

A multivariate polynomial regression (MPR) was used to determine the sensor error (dependent variable) as a function of the four independent variables (threshold, debounce time, debounce steps, speed) using Cecen’s multivariate polynomial regression function [15]. First- through fifth-order MPRs were tested. The R-squared and cross-validated mean absolute error (CVMAE) were used to determine which power produced the best fit that was not rank deficient. Accuracy of the fit was then tested by randomly assigning 90% of the data to train the model, with the remaining data used to test the accuracy through root-mean-square error (RMSE). The difference between the RMSE of the training data and the testing data was calculated to identify over/underfitting (Fit). Whether Fit was positive or negative determined if there was suspected over- or underfitting, respectively.

### 2.5. Data Analysis

Error was calculated for each integer across a range of cadences from 30 to 110 steps/min for each unique combination of settings. To identify which combination of settings produced the lowest error, the standard deviation across a range of cadences was calculated. 

Minimizing the magnitude of error required identifying setting combinations that produced errors within a ±10% range, which is comparable to errors found in a review by Bassett et al. [16]. To identify combinations within that range, the average error (AE) of each grouping was calculated. From these two metrics, a weighted composite score (Equation (1)) was generated, where w was the weights for AMD and (1 − w) was the weight for STD. The lowest composite score was then used to determine which sensor setting was appropriate for the desired range of cadences.

(1) Composite Score=w×AE+(1−w)×STD

This calculation was performed for three ranges of cadences that correlate with walking speeds related to disabled low-mobility ambulators (30–60 steps/min), disabled high-mobility ambulators (30–90 steps/min), and healthy individuals (30–110 steps/min) [17,18,19].

## 3. Results and Discussion

### 3.1. Model Accuracy

The best model was the fourth order polynomial, with an R^2^ = 0.8 and CVMAE of 3.289 (Table 1). The differences between the RMSEs of the testing and training datasets were calculated and reported as Fit (Table 1). Although this model reported a slight overfitting in the increased CVMAE value from the third- and fourth-order polynomial, the 7.8% increase in the R-squared value was deemed an acceptable increase for using the fourth-order polynomial. This model was used to calculate the subsequent error profiles. 

### 3.2. Model Application

The regression model was specifically crafted to generate a set of optimal settings aimed at minimizing the standard deviation of error across the range of cadences while concurrently upholding a variance of ±10% error. Within each cadence range of interest (30–60, 30–90, 30–110), multiple settings met the minimum requirements. This next section defines the sensor setting configuration that secured the lowest variability, recognizing potential alternative combinations.

### 3.3. Disabled and Low-Mobility Ambulator Cadences (30–60 Steps/min)

**Figure 4:**
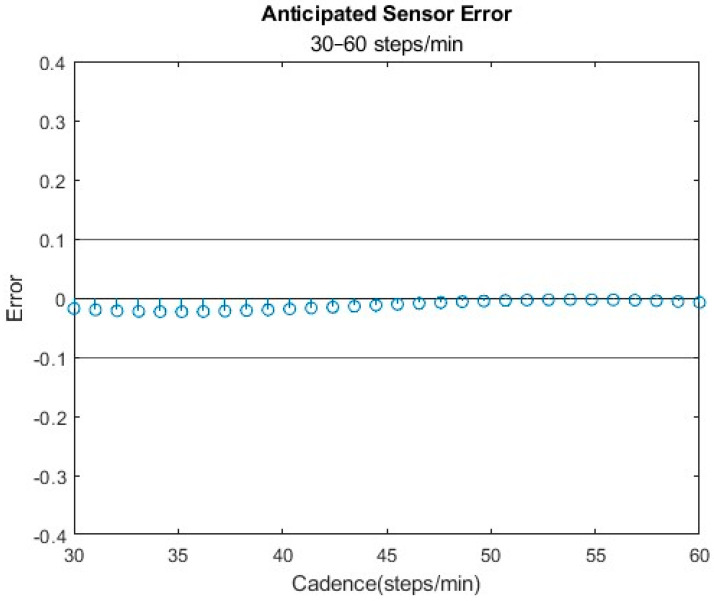
A threshold of 0.48 gs and debounce time of 2240 ms was the recommended setting for a low variance in error across the proposed range.

The optimal sensor configuration, characterized by a threshold of 0.48 g, a debounce time of 2240 ms, and a minimum step count of 6 steps, was determined to be the most accurate for slower cadences. This setting consistently kept the error within ±10% across the specified range of interest (Figure 4). Among the total of five sensor combinations assessed, all fell within the acceptable variance range (±10%) centered at 0 error. 

#### 3.3.1. Threshold

The recommended threshold setting for 30–60 steps/min is 0.48 g, which is lower than the anticipated accelerations of the shank at foot strike by about 50% [8]. It should be noted that the threshold needs to be surpassed to increment step counts and that accelerations of the shank and ground reaction forces are positively correlated with walking speed [19]. It is important to consider the population being studied, as this setting might be too low for certain groups. For instance, children with spastic cerebral palsy may generate high accelerations during spastic movements, potentially counting them as steps. Adjustments, such as increasing the threshold or the minimum required steps, may be necessary. Conversely, amputees often exhibit gait asymmetry, resulting in smaller ground reaction forces from their prosthetic limb, as observed by A. Abouhossein et al. [20].

#### 3.3.2. Debounce Time

When examining slower walking speeds, like 30 steps per minute, it is expected that one step will occur approximately every 2 s. To accurately capture a grouping of steps at or below 30 steps/min, a debounce time equal to or above 2000 ms is necessary. This requirement is reflected in the recommended debounce time identified in the regression analysis.

However, it is important to recognize that individuals, particularly those with a disability, may not walk at a perfect and consistent frequency. As a result, the suggested debounce time should be increased to 2240 ms to accommodate variability in human walking patterns. This adjustment should increase sensor accuracy at lower speeds, which is notoriously less accurate [2,6]. 

### 3.4. Disabled and High-Mobility Ambulator Cadences (30–90 Steps/min)

**Figure 5:**
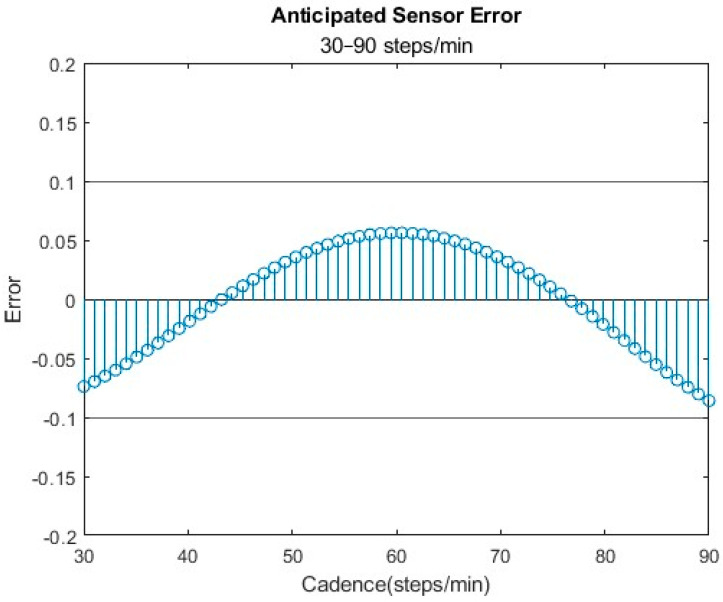
Sensor setting of 0.448 gs and a debounce time of 1920 ms and the negative error that is anticipated at 30–40 steps/min due to the decreased debounce time.

The optimal sensor configuration, characterized by a threshold of 0.448 g, a debounce time of 1920 ms, and a minimum step count of 6 steps, was determined to be the most accurate for disabled and high-mobility individuals. This setting consistently kept the error within ±10% across the specified range of interest (Figure 5). Among the total of six sensor combinations assessed, all fell within the acceptable variance range (±10%) but were not all centered about 0.

#### 3.4.1. Threshold

Like their low-mobility counterparts, disabled high-mobility ambulators present with gait abnormalities that may inhibit their ability to walk long distances, but they can reach higher cadences. It is known that ground reaction force is positively correlated with walking speed; thus, peak impulses would be expected to increase [8]. The model recommended a modest decrease in the threshold of 0.048 g. While this reduction is minimal in the context of the forces involved in walking, it suggests the possibility of multiple sensor combinations being suitable for a desired range of walking speeds.

#### 3.4.2. Debounce

As mentioned above, a debounce time of 2000 ms is required to accurately collect steps that are occurring at or below 30 steps/min. Contrary to this requirement, the proposed algorithm suggested utilizing a debounce sensor setting of 1940 ms. This lower debounce setting could be due to the algorithm’s focus on minimizing the variance in error instead of maximizing sensor accuracy at lower speeds. 

### 3.5. Healthy Ambulators (30–110 Steps/min)

**Figure 6:**
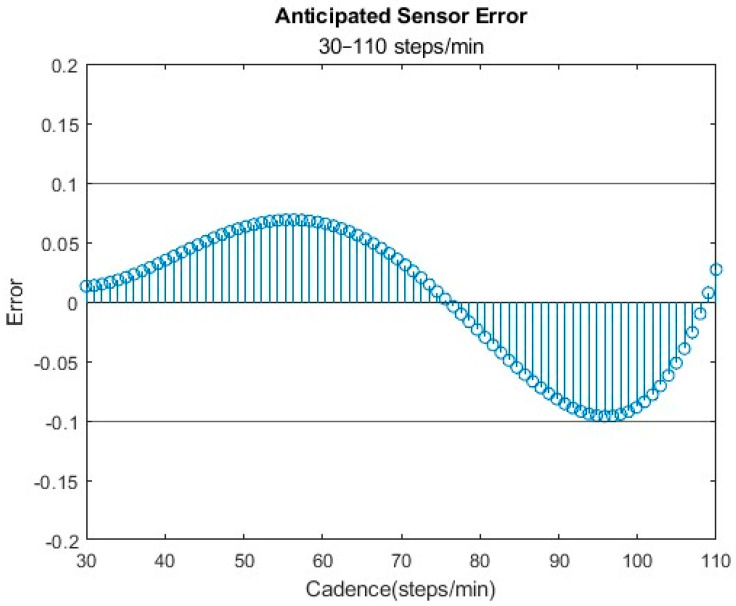
Recommended setting combination of a 0.48 g threshold and 2000 ms of debounce.

The optimal sensor configuration, characterized by a threshold of 0.48 g, a debounce time of 2000 ms, and a minimum step count of 6 steps, was determined to be the most accurate for healthy individuals. This setting consistently kept the error within ±10% across the specified range of interest (Figure 6). Among the total of six sensor combinations assessed, all fell within the acceptable variance range (±10%), but they were not all centered about 0.

#### 3.5.1. Threshold

The recommended threshold setting for a healthy population is very close to the disabled and high-mobility ambulators, at 0.44 gs. As mentioned above, gait speed is positively correlated with ground reaction forces [8]. Although one might expect the threshold to increase with higher cadences, the incorporation of slower cadences within the range of interest requires the threshold to stay at the maximum level for the detection of those slower cadences. This consideration reflects the complexity of setting a threshold that accommodates a broad range of cadence values, including both slower and faster walking speeds. 

#### 3.5.2. Debounce

The suggested debounce time of 2000 ms for healthy individuals is close to that recommended for disabled high-mobility ambulators. This shorter debounce time is suitable for incorporating 30 steps/min cadences. However, longer debounce times come with a potential drawback—they may allow peaks caused by human noise such as weight shifting or fidgeting to be incorrectly counted as steps. These errors are unfortunately residual of the sensors’ onboard processing of acceleration data. Onboard processing has set thresholds for acceleration peaks and the time between peaks that are inflexible, whereas Fortune et al. has shown that flexible timing thresholds can result in more accurate step counts [12]. At this level of processing, it is unfortunately not feasible for the wearable sensor to dynamically change its debounce time due to power demands. One option is to decrease the debounce time to limit the errors caused by human noise such as weight shifting and fidgeting. But this results in slower cadences being undetected, as the time between steps will be greater than the smaller debounce time. For example, if the debounce time were to be lowered to 1 s, any cadence below 60 steps/min would not be detected, as the time between steps is longer than the debounce time. Depending on the experimental design, it will be necessary to assess whether identifying lower cadences takes precedence over preventing additional weight shift peaks from being potentially counted as steps. 

### 3.6. Steps

For each sensor setting recommendation, the algorithm consistently proposed a debounce step count of six steps. The purpose of the debounce step is primarily to remove small bouts of movement that surpass the threshold to be considered a step but do not have a rhythm that is representative of steps. Given that the continuous stepper lacks the inherent variability present in human movement, we believe this recommendation is residual of the method for simulating human steps with a mechanical system. It is important to consider that Orendurff et al. has demonstrated that short walking bouts of less than 12 ± 1 steps account for 40% of daily walking bouts, with 75% of bouts being less than 40 ± 1 steps [21]. Considering that 17% of total bouts consist of 4 ± 1 steps, the minimum step threshold should not fall below 4 steps. This configuration should, in theory, effectively filter out erroneous “steps” induced by weight shifts, fidgeting, standing, and transfers.

### 3.7. Limitations

In the development of this method, it was not intended for the continuous stepper to replicate the inherent variability in stride time observed in healthy subjects. It is crucial to acknowledge that this lack of cadence variability could potentially impact the accuracy of the recommended debounce time, particularly considering that stride time variation tends to increase with decreased cadence [7]. As the steps that were emulated are not perfect representations of human variability, the proposed model may not be the perfect representation of human walking. However, the goal of this proposed method is to provide a procedure to rapidly identify the combinations of sensor settings that provide the most accurate data. This baseline can then be used to initiate human subject testing to finalize the sensor settings. Future variations of the mechanical analog system could focus on emulating the variability through either mechanical manipulation of the cam and piston system or programing cadence variation into the motor speed. A second weakness of this method is that the impact is caused by gravity alone. Any decrease in accelerations at lower cadences may not be emulated properly. This will need to be determined by future work. If there is a speed-dependent change in impact, then this proposed method would benefit from changing the impact material below the pistons so that slower cadences impact a softer surface. This would, in theory, decrease acceleration rates and better model the mechanics of slower human gait.

### 3.8. Implications

Takayanagi et al. and Carcreff et al. have demonstrated that walking speeds collected in a laboratory environment are higher than the average walking speed throughout the day in healthy adults and in children with cerebral palsy, respectively [22,23]. From these findings, it can be inferred that the validation of sensors in a laboratory environment may have an increased likelihood of overlooking slower walking speeds during the calibration process. On the other hand, the average walking speed in the world is decreasing as healthcare improves and life expectancy increases. Accuracy at these slower speeds is considerably lower and requires researchers to put extra attention into determining ideal sensor settings for the highest obtainable accuracy.

The current method for the validation of sensor settings is primarily limited by time, in that multiple settings cannot be tested during human validation trials. This proposed method, when utilized prior to human validation, can identify groups of settings that may produce less error at lower cadences and ensure that the human validation process is efficient and targeted. By implementing this new method before human validation, researchers can streamline the calibration process and enhance the overall accuracy and applicability of sensor settings across diverse walking conditions, particularly at slower walking speeds, which are hardest to collect accurately.

It should also be noted that wearable sensors are constantly balancing processing power with power consumption. Many methods for determining step count are dependent on raw acceleration data collected at high frequency and mounted at locations on the body that are unnatural, like the ankle and thigh [2,3,11]. These sensors commonly have a battery life of a few days, which is not useful in longitudinal studies or for tracking activity changes over time. Onboard processing of data using acceleration thresholds is the most cost-efficient method of step counting that minimizes power draw and processing power while maximizing collection time. Our proposed model focuses on sensors that could be used for multiple weeks to collect data through onboard processing, which are more cost-efficient for clinics or studies looking at overall activity. To maximize accuracy in these studies, it is pertinent to optimize sensor configuration to the anticipated population of interest.

This method enables the reduction of the sensor setting matrix space to a manageable subset, which can then be fine-tuned through human subject validation. Notably, it allows researchers to identify settings with consistently high error across a range of interest, providing confidence in the observed changes during daily activities, even if an exact step count is not crucial (e.g., for studying variable cadence). Alternatively, for those prioritizing accurate step counts, the method helps identify settings with low combined errors. This flexibility enhances the adaptability and precision of sensor settings for diverse walking conditions.

## 4. Conclusions

Wearable sensors are being used more frequently by researchers interested in quantifying human movement in the free-living environment. Unfortunately, the accuracy of wearable sensors is notoriously low, particularly at slower cadences. Current methods to validate sensor accuracy compare human step counts recorded by an observer and sensor counts collected in a controlled environment. These methods are limited by time and minimize the ability to explore a vast array of different sensor setting combinations and walking speeds. In this paper, we have described a method to emulate steps and model the anticipated error across the subspace of sensor setting combinations. With this novel model, researchers can identify sensor settings prior to human validation to ensure the proper setting combinations are being examined during validation.

## Figures and Tables

**Table 1:** Resulting R-squared, RMSE, CVMAE, and fit of trained models with first- to fifth-order polynomials. The fourth-order polynomial was determined to produce the highest accuracy while accounting for overfitting, which increases in the fifth order.

	1st Order	2nd Order	3rd Order	4th Order	5th Order
R-Squared	0.271	0.524	0.722	0.800	0.847
RMSE	0.086	0.0813	0.075	0.044	0.185
CVMAE	5.085	5.295	3.383	3.589 ^1^	4.171
Fit	−0.062	−0.012	0.019	0.003	0.154

^1^ The CVMAE of the fourth-order polynomial is higher than the third order, suggesting overfitting from the third to fourth order. The fit suggests very little overfitting when taking training and testing data into account.

## Data Availability

The data supporting the conclusions of this article will be made available by the authors on request.
